# Traditional medicine in a modern light: a scoping review on the osteoprotective potential of *Eucommia ulmoides*

**DOI:** 10.3389/fphar.2025.1673870

**Published:** 2026-01-21

**Authors:** Yuanzhong Wang, Guiju Chen, Qin Wang, Bilin Liu, Qi He, Xia Huang, Kok-Yong Chin

**Affiliations:** 1 Department of Pharmacology, Faculty of Medicine, Universiti Kebangsaan Malaysia, Kuala Lumpur, Malaysia; 2 Chongqing Chemical Industry Vocational College, Chongqing, China; 3 Xiangyang Central Hospital, Affiliated Hospital of Hubei University of Arts and Science, Xiangyang, Hubei, China; 4 The First Affiliated Hospital of Chongqing Medical and Pharmaceutical College, Chongqing, China

**Keywords:** Du-Zhong, osteoclast, osteoblast, osteopenia, traditional Chinese medicine

## Abstract

**Background/Objectives:**

*Eucommia ulmoides* (Du-Zhong), a traditional Chinese medicinal plant, has long been valued for its ability to strengthen bones and muscles. Recent research highlights its potential in supporting bone health and managing osteoporosis. This scoping review summarises existing evidence on the role of *Eucommia ulmoides* in osteoporosis prevention and treatment.

**Methods:**

A comprehensive literature search was conducted from inception to November 2025 across six databases: PubMed, Scopus, Web of Science, CNKI, Wanfang and CQVIP. Studies investigating the effects of *Eucommia ulmoides* extracts or its bioactive compounds on bone-related outcomes in cellular, animal, or human models of osteoporosis were included.

**Results:**

Ninety studies met the inclusion criteria. The evidence demonstrates that *Eucommia ulmoides* and its bioactive compounds, most notably aucubin, geniposide, rutin, and pinoresinol diglucoside, consistently promote osteoblastogenesis and inhibit osteoclastogenesis. These effects are mediated through the modulation of key signalling pathways, including BMP/SMAD, Wnt/β-catenin, OPG/RANKL, and MAPK/NF-κB. In animal models, the treatment improved bone mineral density (BMD), enhanced trabecular microarchitecture (increased bone volume fraction, trabecular number and thickness), and increased bone biomechanical strength. Clinical studies, while preliminary, reported that *Eucommia ulmoides*-based formulations (e.g., Quanduzhong capsules, Eucommia granules) were associated with improved BMD, reduced bone resorption markers, and alleviated clinical symptoms in osteoporotic patients.

**Conclusion:**

This scoping review consolidates a substantial body of evidence supporting the osteoprotective potential of *Eucommia ulmoides*. Its multi-targeted mechanism of action, targeting both bone formation and resorption, positions it as a promising candidate for the management of osteoporosis. The findings underscore the necessity for future research to prioritise standardised extract preparation, detailed pharmacokinetic studies, and rigorous, large-scale clinical trials to definitively establish its efficacy and safety in humans.

## 1 Introduction

Osteoporosis is a prevalent skeletal disorder affecting millions of older adults globally. It is characterised by reduced bone mineral density (BMD) and the deterioration of bone microarchitecture, leading to an increased risk of fragility fractures, particularly in weight-bearing bones such as the spine, hip, and wrist. These fractures significantly affect morbidity, quality of life, and mortality, especially among the elderly (Chin et al., 2022). The socioeconomic burden of osteoporosis is significant, resulting in increased healthcare costs, long-term disability, and a growing demand for rehabilitation and caregiving services (Singer et al., 2023). With postmenopausal women at greater risk due to rapid bone loss caused by oestrogen deficiency, osteoporosis-related fractures represent a significant public health concern (Mohamad et al., 2020).

Despite advances in pharmacological treatments, managing osteoporosis remains a complex challenge. Standard therapies such as bisphosphonates, selective oestrogen receptor modulators, hormone replacement therapy, and newer agents like denosumab and teriparatide have demonstrated efficacy in slowing bone loss and reducing fracture risk (Cao et al., 2024; Li et al., 2023). However, these treatments are not free from side effects, and long-term adherence is often poor (Ayers et al., 2023; Compston et al., 2019). The pursuit of better treatment for osteoporosis is still ongoing.

Given these challenges, there is growing interest in exploring alternative approaches, particularly those rooted in traditional Chinese medicine (TCM). With a history spanning over 2,000 years, TCM offers a holistic approach to health by addressing the root causes of imbalances in the body’s organ systems (Wang X. et al., 2024). In terms of bone health, TCM herbal formulations have been used to strengthen bones, nourish the kidneys, and improve musculoskeletal function. This application is based on the TCM belief that bone health is closely connected to kidney function (Bao, 2024).

Among the various TCM herbs, *Eucommia ulmoides* (EU) is well recognised for its benefits to bone health and has a long history of treating bone-related conditions. Native to China, EU has been traditionally used to tonify the liver and kidneys, strengthen muscles and bones, and alleviate symptoms such as lower back pain, joint stiffness and fatigue, which are common indicators of bone deterioration (Bao, 2024; He et al., 2014; Wang C. Y. et al., 2019). Modern pharmacological research supports these traditional uses, suggesting that EU exhibits both bone anabolic and anti-resorptive effects, thereby reducing the risk of osteoporosis by stimulating osteoblast activity and inhibiting osteoclast function (He et al., 2014; Wang C. Y. et al., 2019; He et al., 2019).

EU contains an array of bioactive compounds believed to contribute to its beneficial effects on bone health. Among the most notable bioactive compounds are polysaccharides (Cao et al., 2024; Yu et al., 2024; Li et al., 2024a), lignans (Han et al., 2022; Gu et al., 2011; Fujiwara et al., 2016), flavonoids (Zhai et al., 2021; Zhu, 2024), iridoids (Takamura et al., 2007; Zhu and Sun, 2018) and phenolic acids (Zhang et al., 2020; Liang et al., 2023). Each of these bioactive compounds exhibits pharmacological activities relevant to bone metabolism. For instance, Eucommia polysaccharides and phenolic acids (e.g., chlorogenic acid) can protect bone tissue by reducing oxidative stress and inflammation (Zhang et al., 2020; Liang et al., 2023). Lignans [e.g., pinoresinol diglucoside (PDG)] (Han et al., 2022; Gu et al., 2011; Fujiwara et al., 2016) and iridoids [e.g., geniposidic acid (GA) and aucubin] (Takamura et al., 2007; Zhu and Sun, 2018) have been shown to stimulate osteoblastic bone formation process and inhibit osteoclastic bone resorption. Flavonoids in EU promote osteoblast proliferation and differentiation, which are critical for increasing bone formation (Zhai et al., 2021; Zhu, 2024). Together, these bioactive compounds work through various molecular mechanisms to support bone health, positioning EU as a promising candidate for the prevention and treatment of osteoporosis (Liang et al., 2023; Wang et al., 2022; Mai et al., 2024; Zuo et al., 2024).

This review aims to systematically explore the current literature on the role of EU in osteoporosis management. It will focus on the plant’s effects on bone metabolism and the key regulatory mechanisms involved. By reviewing both preclinical and clinical studies, this paper seeks to provide a comprehensive understanding of how EU may contribute to osteoporosis prevention and management and to offer insights into its therapeutic potential in integrative medicine.

## 2 Materials and methods

### 2.1 Literature review

This scoping review was conducted following the guidelines outlined by Arksey and O'Malley (Arksey and O'Malley, 2005) and in accordance with the PRISMA extension for scoping reviews (Tricco et al., 2018). The protocol of the study is registered with the Open Science Foundation (url: https://osf.io/3p4hw/). The primary aim of this review was to explore the role of EU in osteoporosis management by systematically analysing relevant studies. The following steps were undertaken: (1) identifying the research question, (2) identifying relevant studies, (3) selecting studies, (4) charting the data, and (5) collating, summarising, and reporting the results.

### 2.2 Identifying the research question

The primary research question for this scoping review was: What are the roles of EU in the management of osteoporosis? This review considered the therapeutic effects of EU on bone health and metabolism, including its effects on bone formation and resorption. Additionally, the review aimed to elucidate the underlying mechanisms, particularly the regulation of key signalling pathways, such as the Wnt/β-catenin and receptor activator of nuclear factor kappa-Β (RANK) ligand (RANKL)/osteoprotegerin (OPG) pathways, which are crucial in maintaining bone homeostasis.

### 2.3 Identifying relevant studies

A systematic literature search was conducted in November 2025 across three international databases (PubMed, Scopus and Web of Science) and three major Chinese databases [China National Knowledge Infrastructure (CNKI), Wanfang Data and the VIP Chinese Science and Technology Periodicals Database (CQVIP)] to ensure comprehensive coverage. The search strategy employed the search string (“Du-Zhong” OR “Du Zhong” OR “Eucommia ulmoides”) AND (osteoporosis OR bone OR skelet* OR osteoblast* OR osteoclast* OR fracture*) within the title or abstract fields. For the Chinese databases, the search string “杜仲” AND “骨质疏松” was used within the title and abstract fields.

Studies published in English and Mandarin that evaluated the effects of EU on bone-related outcomes were included, regardless of study design. Both *in vitro*, *in vivo* studies and clinical trials on this topic were included. Network pharmacology studies without laboratory validation were excluded. All articles without primary data, such as reviews, editorials, letters, commentaries and perspectives, were excluded. Conference abstracts were excluded due to incomplete data and possibly overlapping with full articles. Studies involving compound preparations of EU were excluded because the effects of the plant alone cannot be delineated. No additional filters, such as publication date, were applied during the search.

### 2.4 Study selection

The search data were downloaded from each database and merged using EndNote (version 21.2, Clarivate, London, UK). Duplicated records were removed electronically and manually. Five independent reviewers (GJC, QW, BLL, QH and XH) screened the titles and abstracts to identify studies that met the inclusion criteria. Full-text articles of the selected studies were then retrieved and assessed for eligibility. In cases where consensus could not be reached, other authors (KYC and YZW) were consulted to finalise the selection.

### 2.5 Data charting

The selected studies were organised using a standardised data extraction table, which captured details such as authors, publication year, animal or cell models used, plant parts used, dosage, treatment duration, skeletal effects and mechanisms. Data extraction was performed by two authors (GJC and YZW), and discrepancies were resolved through discussion.

### 2.6 Data synthesis

The findings from the literature were summarised and narrated qualitatively due to the heterogeneity of study design and outcomes measured. Quantitative synthesis of outcomes was not performed.

## 3 Results

### 3.1 Article selection

**FIGURE 1:**
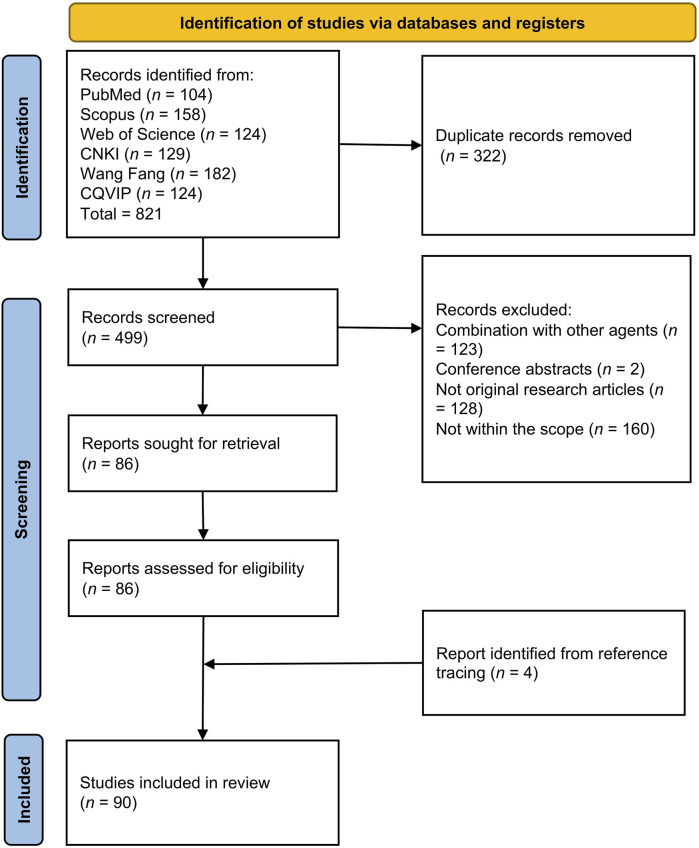
PRISMA flowchart showing the selection of articles. Abbreviations: CNKI, China National Knowledge Infrastructure; CQVIP, the VIP Chinese Science and Technology Periodicals Database.

A comprehensive literature search across PubMed, Scopus, and Web of Science initially identified 821 unique articles. After removing duplicates (*n* = 322), a total of 499 articles were subjected to screening. Of these, 413 articles were excluded for the following reasons: (1) lack of primary data (*n* = 128); (2) not within the scope of this review (*n* = 160); (3) involving formulations or multi-herbal mixtures (*n* = 123); (4) conference abstracts (*n* = 2). Additionally, four relevant articles were identified through reference tracing. Ultimately, 90 articles met the inclusion criteria and were included in this review. The article selection process is summarised in Figure 1.

### 3.2 Study characteristics

The analysed body of evidence comprised 90 studies investigating EU and its derivatives for osteoporosis management. Research was broadly categorised by the specific bioactive compounds investigated, the plant parts utilised for extraction, and specific formulations (Table 1).

**TABLE 1:** Classification and key compounds/extracts of *Eucommia ulmoides* studied.

Category	Subcategory	Number of studies	Key compounds/Extracts (references)	CAS
Bioactive Compounds	AU	8	AU (Rao, 2004; Li et al., 2022; He et al., 2023b; Huang et al., 2024; Wang et al., 2024b; Zheng, 2025b; Huang et al., 2022; Lin et al., 2011)	479–98–1
	PDG	2	PDG (Arksey and O'Malley, 2005; Song et al., 2023)	63,902–38–5
	GEN	4	GEN (Chen, 2015; Xiao et al., 2022; Xiao et al., 2023a; Xiao et al., 2023b)	24,512–63–8
	5-HMF	2	5-HMF (Zhang et al., 2016; Lang et al., 2025)	67–47–0
	Rutin	2	Rutin (Tan et al., 2014; Zhang, 2022)	153–18–4
	Chlorogenic Acid	1	Chlorogenic Acid (Li et al., 2024b)	327–97–9
	Kaempferol	2	Kaempferol (Liu et al., 2023; Zhang, 2022)	520–18–3
	Quercetin	3	Quercetin (Chen, 2015; Xiao et al., 2019; Zhang, 2022)	117–39–5
	Geniposidic Acid	3	Geniposidic Acid (Rao, 2004; Shao, 2022; Lin et al., 2011)	27,741–01–1
	β-Sitosterol	1	β-Sitosterol (Liu, 2024)	83–46–5
	Pinoresinol	1	Pinoresinol (Tian et al., 2018)	487–36–5
Plant Parts and Extracts	Dry Bark (Cortex)	26	DZCE (Qi et al., 2019; Shen et al., 2024; Yang et al., 2022), EE-EU (Pan et al., 2014), EUE (Ha et al., 2003; Xing and Feng, 2022; Zheng, 2025a; Zhang, 2011; Weng, 2014; Zhang, 2010b; Cai, 2009; Tong, 2009; Luo, 2016; Zhang, 2009a; Xiong and Zhao, 2016; Xiong, 2020; Zhao and Chen, 2019; Zhao et al., 2017; Raisz, 2005; Zhao et al., 2020a)	mixture
			EuOCP3 (Song et al., 2024; Ho et al., 2024), TL (Li et al., 2018b), Total Flavonoids (Bark) (Li et al., 2010), Pinoresinol Diglucoside and Pinoresinol (Tian et al., 2018)	
​	Leaves	19	ELE (Zhang, 2010b; Rao, 2004; Ma et al., 2024; Zhang, 2003; Dai, 2012; Liu, 2018; Du et al., 2023; Hu and Wang, 2002; Zhang et al., 2024a; Zhang et al., 2024b; Li et al., 2011; Zhang et al., 2014), EF (Yi, 2024)	mixture
			TFEL (Yin et al., 2025; Zhang et al., 2012; Hu, 2018; Jiang, 2024)	
​	Seeds	3	TGEUS (Zhou et al., 2021; Xiao, 2008; Liu, 2012)	mixture
Other Extracts and Formulations	Formulations	11	QDZ (Li et al., 2024c; Zhang, 2009b; Sun and Wu, 2022; Wu et al., 2024; Wang and Sun, 2022; Cai, 2021; Wang, 2018; Liang et al., 2017),EU Granules (Yang et al., 2023; Tang, 2012; Wang and Chu, 2014)	mixture
​	Polysaccharides	2	EuOCP3 (Song et al., 2024; Ho et al., 2024)	mixture
​	Targeted Conjugates	1	SGPA (Yuan, 2018)	mixture

Abbreviations: 5-HMF, 5-(hydroxymethyl)-2-furaldehyde; AU, aucubin; DZCE, Du-Zhong cortex extract; EE-EU, ethanol extracts of *Eucommia ulmoides*; EF, eucommia flavone; ELE, *Eucommia* leaf extract; EU, *eucommia ulmoides*; EuOCP3, polysaccharide purified from *Eucommia ulmoides* Oliver; EUE, *Eucommia ulmoides* extracts; GEN, geniposide; PDG, pinoresinol diglucoside; QDZ, quanduzhong capsule; SGPA, Ser-Asp-Ser-Ser-Asp peptide-geniposidic acid conjugate; TGEUS, total glycosides from *Eucommia ulmoides* seed; TFEL, total flavonoids from *eucommia ulmoides* leaves; TL, total lignans.

Aucubin was identified as the most commonly studied single compound. Research on plant parts highlighted dry bark and leaves as the primary sources of therapeutic extracts, with a significant number of studies focusing on total flavonoids and glycosides. Additionally, specific formulated products such as the Quanduzhong capsule and polysaccharides were investigated. The methodologies employed across the studies were diverse, encompassing *in vitro*, *in vivo*, and clinical research, which collectively underscored the broad osteoprotective potential of EU.

In terms of methodology, a substantial number of studies were primarily cell-based, focusing on processes of osteoblastogenesis and osteoclastogenesis. Animal studies formed the majority, primarily conducted in rodent models of ovariectomy (OVX)- and glucocorticoid-induced osteoporosis (GIOP). Several studies employed integrated *in vitro* and *in vivo* approaches to elucidate the mechanisms of action. Several clinical trials investigating the efficacy of various EU-based formulations in improving BMD, bone metabolism markers, and clinical symptoms in osteoporotic patients were identified.

### 3.3 The effects of *Eucommia ulmoides* extract in osteoporosis treatment

*Eucommia ulmoides* extracts (EUE) were consistently shown to exert pro-osteogenic actions across cellular and animal models. In osteoblastic cell lines, differentiation was promoted by EUE across a wide concentration range, largely through the upregulation of Runx2, Osterix and other osteogenic markers, while osteoclastogenesis was suppressed via modulation of RANKL-OPG signalling, without detectable cytotoxicity (Lee et al., 2016; Liang et al., 2017). Bioactive constituents such as geniposide, aucubin and gallic acid were identified, with chloroform fractions displaying the strongest stimulatory effects on pituitary growth hormone release and osteoblast-like cell proliferation (Ha et al., 2003). Collectively, these findings indicated that osteoblast activity was directly enhanced by multiple E. ulmoides-derived components.

Pro-osteogenic effects were also observed in mesenchymal stem cells. In rat bone marrow mesenchymal stem cells (BMSCs), osteopontin (OPN) expression was increased by aqueous and methanol bark extracts, whereas OPG remained largely unchanged, suggesting an OPN-centred mechanism (Zhang Y. H., 2010). Lineage commitment was further influenced by suppression of adipogenic markers, particularly fatty acid-binding protein (FABP), even when classical osteogenic transcription factors were not markedly induced, thereby indicating an indirect promotion of osteogenesis via inhibition of adipogenesis (Xian, 2010). More recently, EUE drove proliferation and osteogenic differentiation in human and murine BMSCs through the Nur77/MDM2/p53 axis; the effect was abolished by Nur77 knockdown and restored by p53 inhibition, confirming the essential role of this pathway (He et al., 2023a). In primary rat osteoblasts, BMP2 gene expression was significantly upregulated in a concentration-dependent manner following short-term exposure to water extracts, although prolonged treatment attenuated this response (Xing and Feng, 2022).

Animal studies further supported the osteoprotective potential of EUE. In OVX-induced osteoporosis, long-term administration of EU cortex decoctions improved trabecular microarchitecture, increased BMD and elevated osteogenic markers, accompanied by reduced TRACP and enhanced antioxidant status (Luo, 2024). Alveolar bone density and Wnt/β-catenin signalling were likewise improved in OVX rats treated with EUE (Zheng L., 2025), while other studies reported decreases in bone resorption surfaces and increases in trabecular bone volume via RANKL suppression (Zhang, 2011). Benefits were also observed when EUE was administered alone or in combination with exercise (Lin, 2018), and processed forms such as salt-roasted extracts demonstrated stronger anti-osteoporotic effects (Weng, 2014).

Beyond post-ovariectomy models, EUE alleviated bone loss in glucocorticoid-induced osteoporosis by reducing urinary calcium and phosphorus, improving BMD and biomechanical properties, and enhancing BMP-2 expression (Zhou, 2016). Disuse-related bone deterioration in hindlimb-suspended rats was prevented by EU treatment, which maintained bone strength and trabecular structure while suppressing bone turnover (Pan et al., 2014). In lead exposure-related osteoporosis, EUE restored BMD, improved mineral homeostasis, normalised alkaline phosphatase (ALP), osteocalcin and RANKL levels, and increased OPG, leading to recovered trabecular volume and reduced marrow adiposity (Qi et al., 2019). In diabetic osteoporosis, aqueous EUE improved glycaemic control, preserved bone microarchitecture and upregulated Runx2 and BMP-2, largely through the Nrf2/HO-1 antioxidant pathway and maintenance of calcium balance (Shen et al., 2024).

The osteogenic potential of EU was consistently demonstrated across studies using BMSCs and osteoblast cell lines. Treatment of human and rat BMSCs with the aqueous extract (10 mg/mL) during lineage induction enhanced proliferation, suppressed apoptosis, and shifted differentiation towards an osteogenic phenotype. These effects were associated with increased ALP activity, greater mineral deposition, and upregulation of osteogenic markers, alongside reductions in adipogenic proteins. Mechanistically, Nur77 and MDM2 were upregulated and p53 ubiquitination was increased, indicating that the Nur77/MDM2/p53 axis mediated the pro-osteogenic response. Nur77 knockdown reversed these effects, which were subsequently restored by p53 inhibition (He et al., 2023a).

Water and ethanol extracts of EU bark similarly promoted osteogenic commitment of rat BMSCs, as reflected by increased Runx2 expression and suppression of fatty acid-binding protein, although Osterix expression was generally reduced. Adipogenic signalling appeared to be particularly inhibited, suggesting that lineage allocation was shifted away from adipogenesis (Zhang X., 2010).

Findings from osteoblast cell line studies aligned with those from BMSC models. Ethanol extract of EU cortex increased MC3T3-E1 proliferation and ALP activity, whilst downregulating RANKL and modestly elevating OPG, thereby enhancing the OPG/RANKL ratio and indirectly suppressing osteoclastogenic signalling (Xu, 2013).

Further work on the Wnt pathway demonstrated that bark extract (1/1,000 dilution) increased Fzd2/3 and β-catenin expression while downregulating WIF1 during BMSC osteogenic induction. These changes suggested that canonical Wnt signalling contributed to extract-induced osteogenesis (Zhang, 2012).

BMSCs derived from ovariectomised rats responded to ethanol extract treatment with enhanced proliferation, higher mineralisation rates, and upregulation of RhoA/ROCK pathway components together with increased expression of OPN, Runx2, and OCN. These findings indicated that osteogenic differentiation was facilitated through activation of RhoA/ROCK signalling (Lin, 2019).

Across numerous rodent models, EU was consistently shown to mitigate osteoporotic bone loss and improve bone metabolism. In OVX and glucocorticoid-induced osteoporosis, BMD, oestradiol levels, bone turnover markers, and femoral biomechanical strength were improved when EU ingestion, whole-body vibration, or their combination were applied (Wang and Luo, 2019). Similar osteoprotective effects were observed when EU bark was administered to OVX rats, as increases in mineral content, tibial bending strength, and alkaline phosphatase were recorded, indicating suppressed bone resorption and enhanced bone formation (Cai, 2009). Upregulation of osteogenic mediators, including BMP-2, was also reported *in vivo* and *in vitro*, suggesting stimulation of osteogenic signalling pathways (Zhao, 2009).

Salt-processed EU demonstrated protective actions in retinoic-acid models, where medium and high doses increased BMD, bone mineral content, oestradiol, and multiple bone growth factors, whilst inflammatory cytokines were reduced (Xiong and Zhao, 2020a; Xiong and Zhao, 2020b). In OVX rats, EU treatment increased the expression of osteogenic factors such as TGF-β and FGF2, improved biomechanical strength, and elevated BMD, confirming its regulatory effects on bone metabolism (Feng, 2009; Ge et al., 2009; Tong, 2009). Improvements in trabecular microarchitecture, oestradiol levels, and bone turnover balance were also recorded following medium- and high-dose extract administration, with micro-CT revealing enhanced bone volume, connectivity, and trabecular number (Luo, 2016). Salt-processed EU likewise increased serum oestradiol, reduced bone resorption markers, elevated lumbar BMD, and improved tibial strength over long-term administration (Zhang, 2009a).

Enhancement of fracture resistance and bone structural integrity was further demonstrated, as ethanol and aqueous extracts increased femoral fracture and crushing forces in OVX models (Hou and Wang, 2010). In ageing and D-galactose-induced osteoporosis, EU improved serum IGF-1, increased ALP activity, and raised femoral BMD, suggesting stimulation of bone formation in senile bone loss (Xiong and Zhao, 2016). EU granules exerted additional protective effects through improvements in BMD, trabecular indices, bone strength, and regulation of JAK2/STAT3 signalling (He et al., 2025). Regulatory actions on the OPG/RANKL/RANK axis were also observed, indicating that osteoclastogenesis was suppressed and bone formation was promoted, with efficacy exceeding that of alfacalcidol in some parameters (Yang et al., 2023).

Beyond oestrogen-deficiency models, favourable effects were recorded in diabetic osteoporosis, where EU restored bone microstructure, increased Runx2 and BMP-2 expression, reduced osteoclast numbers, improved calcium homeostasis, and activated the Nrf2/HO-1 antioxidant pathway (Shen et al., 2024). Anti-inflammatory and bone-turnover-normalising effects were also demonstrated in retinoic-acid osteoporosis, where BMD, OPG, and oestradiol increased and TNF-α, IL-6, PINP, and CTX declined (Xiong, 2020). In long-term OVX studies, high-dose salt-processed EU increased whole-body, femoral, and vertebral BMD and improved ALP, osteocalcin, and TRACP, with efficacy comparable to oestradiol (Zhao and Chen, 2019). Enhanced BMP-2 expression and improved BMD in senile models further supported its osteogenic actions (Zhao et al., 2017).

Oestrogen deficiency represented a major contributor to osteoporosis (Raisz, 2005). In an ovariectomy-induced osteoporosis model, EUE significantly improved the biomechanical properties of the femur, especially at higher doses (300 or 500 mg/kg/day), while preventing BMD loss and enhancing trabecular microarchitecture (confirmed by micro-CT analysis) after 16 weeks of treatment. Furthermore, EUE reduced bone turnover markers such as osteocalcin and ALP without inducing uterine hyperplasia, suggesting its potential as a safe alternative treatment for postmenopausal osteoporosis (Zhang et al., 2009). These findings were validated in another similar study using lower doses of EUE. EUE (50–200 mg/kg/day for 6 weeks) alleviated oestrogen deficiency-induced osteoporosis in castrated rats by improving bone microstructure, reducing femoral cell apoptosis, and regulating serum phosphorus and IL-6 levels (Yang et al., 2022). EUE has also been optimised using response surface methodology. The optimised extract, administered at 4 g/kg in an OVX rat model for 2 months, significantly improved bone quality by increasing calcium, phosphorus, and chromium levels, and enhancing the biomechanical properties of trabecular bone. These results underscore its therapeutic potential in treating osteoporosis (Zhao Z. et al., 2020).

Prolonged immobility can cause significant bone loss (Goropashnaya et al., 2021). In a lead acetate-induced osteoporosis model, EUE administration prevented BMD reduction and improved serum calcium and phosphorus levels. Additionally, EUE lowered elevated ALP, osteocalcin and RANKL levels, while enhancing serum OPG and the OPG/RANKL ratio. Bone histomorphometry analysis revealed that EUE restored bone volume and trabecular thickness, and reduced bone marrow adipocyte size and number, emphasising its ability to stimulate bone formation and inhibit bone resorption in lead exposure (Qi et al., 2019). Table 2 summarises the studies presented in this section.

**TABLE 2:** Summary of experimental studies investigating *Eucommia ulmoides* extracts in osteoporosis models.

Category	References	Model	Treatment	Major findings
*In Vitro* Studies				
Osteoblast/Bone Cell Models	Yi et al. (2024)	Saos-2 cells; Osteoclast co-culture	EUE (0.1–10 μg/mL)	↑ Runx2, Osterix, OPG; ↓ RANKL. Promoted bone formation and inhibited resorption
​	Lin et al. (2011)	Rat pituitary; MG-63/Saos-2 cells; Osteoclast co-culture	EUE (10^−1^-10^–8^ mg/mL)	↑ GH release; ↑ osteoblast proliferation; ↓ osteoclast proliferation
​	Luo (2024)	Rat primary calvarial osteoblasts	Bark water extract (3 × 10^-1^–3 × 10^-5^ mg/mL)	Dose-dependent ↑ BMP2 gene expression
​	Zhang (2012)	MC3T3-E1 osteoblasts	Cortex ethanol extract (10^−1^–10^–3^ mg/mL)	↑ proliferation, ALP activity; ↑ OPG/RANKL ratio
​	Ha et al. (2003)	Osteoblast cell line	EUE (180–540 μg/mL)	↑ ALP, osteocalcin, collagen I, TGF-β1
BMSC Models	Xing and Feng (2022)	Human and rat BMSCs	Aqueous extract (10 mg/mL)	↑ proliferation and osteogenesis via Nur77/MDM2/p53 pathway; ↓ adipogenesis
​	Wang and Luo (2019)	BMSCs from OVX rats	Ethanol extract (50–200 mg/kg *in vivo* conditioning)	↑ mineralization, proliferation; ↑ RhoA/ROCK, OPN, Runx2, OCN.
​	Lin (2019)	Rat BMSCs	Bark ethanol extract (1/1000 dilution)	Activated Wnt/β-catenin pathway (↑ Fzd2/3, β-catenin; ↓ WIF1)
​	Xian (2010)	Rat BMMSCs	EUE (1 × 10^-2^–1 × 10^-5^ dilutions)	↑ OPN expression (methanol extract > water extract)
​	He et al., 2023a; Xu (2013)	Rat BMSCs	Water and methanol extracts (10^−3^–10^–4^)	Promoted osteogenesis by suppressing adipogenesis (↓ FABP)
*In Vivo* Studies				
OVX Model: Microarchitecture and Biomechanics	Yang et al. (2022)	OVX SD rats	EUE (100–500 mg/kg, 16 w)	↑ BMD, BV/TV, Conn.D; ↑ biomechanical strength
​	Zheng (2025a)	OVX SD rats	EUE (0.54–2.16 g/kg, 200 days)	↑ BMD, trabecular structure; ↑ BMP-2, OPG, RUNX2; ↓ TRACP.
​	Zhou (2016)	OVX SD rats	EUE and Salt-roast Extract (4.0 g/kg, 12 w)	↑ BMD; salt-roasted extract showed superior efficacy
​	Xiong and Zhao (2016)	OVX SD rats	Duzhong Extract (0.35–0.56 g/kg, 60 days)	↑ femoral fracture and crushing strength
​	Goropashnaya et al. (2021)	OVX rats	EUE (4 g/kg, 2 m)	↑ serum Ca/P/Cr; ↑ bone ultimate load, stiffness
OVX Model: Bone Metabolism	Xiong (2020)	OVX SD rats	EUE (2.76 g/kg, 12 w)	↑ BMD, strength; activated OPG/RANKL/RANK pathway
​	Zhang (2011)	OVX SD rats	EUE (2.1–4.2 g/kg, 12 w)	↑ alveolar bone formation via Wnt/β-catenin pathway
​	Cai (2009)	OVX + Dexamethasone Rats	EUE ± Whole-Body Vibration	↑ BMD, estradiol; ↓ bone turnover; combination effective
​	Zhang (2009a)	OVX SD rats	EUE (1.5–6 g/kg, 16 w)	↑ BMD, BV/TV, Tb.N; ↑ E2; ↓ bone turnover markers
​	Zhao et al. (2020a)	OVX rats	EUE (50–200 mg/kg, 1.5 m)	Improved trabecular structure; modulated serum Ca/P, IL-6
OVX Model: Processed Eucommia and Formulations	Zhao et al. (2017)	OVX SD rats	Salt-processed Extract (1.5–6 g/kg, 12 w)	↑ BMD (whole-body, femoral, vertebral); improved bone turnover
​	Yang et al. (2023)	OVX SD rats	Eucommia Granules (2.76 g/kg, 12 w)	↑ BMD, BMC, strength; modulated JAK2/STAT3 signalling
​	Zhao (2009)	OVX SD rats	Eucommia Bark (1 g/10 rats/d, 3 m)	↑ femoral mineral content, tibial bending strength, ALP.
​	Hou and Wang (2010)	OVX SD rats	Salt-processed Eucommia (0.33 mg/g/d, 90 days)	↑ lumbar BMD, tibial strength; ↑ E2; ↓ bone resorption markers
​	Tong, 2009; Luo (2016)	OVX rats	Decoction/ethanol Extract	↑ tibial strength, BMD, serum E2, and IGF-I
​	Lin (2018)	OVX Wistar rats	EUE (5.6 g/kg/d, 3 m)	↑ trabecular bone volume; ↓ bone resorption surface and RANKL.
​	Xiong and Zhao (2020a), Ge et al. (2009)	OVX SD rats; MSCs	Bark extracts	↑ expression of TGF-β, FGF2, and BMP-2
Other Disease Models				
GIOP	Pan et al. (2014)	GIOP SD Rats	EUE (100–500 mg/kg/d, 12 w)	↑ BMD, Runx2, BMP2; prevented cartilage degradation via AR.
Diabetic Osteoporosis (DOP)	Zhang (2010b)	DOP C57BL/6 Mice	Aqueous Extract (2.5 g/kg/d, 6 w)	↑ BMD, BV/TV; ↑ Runx2, Bmp2, Nrf2/HO-1
Disuse Osteoporosis	Qi et al. (2019)	Hindlimb-Suspended SD Rats	EUE (300 mg/kg, 6 w)	Prevented bone loss; ↑ BMD, Conn.D, Tb.Th
Senile Osteoporosis	He et al., 2025; Raisz (2005)	D-galactose-Induced Rats	Salt-processed Cortex (1.5–6 g/kg, 4 w)	↑ femoral BMD; ↑ BMP-2 expression
Lead Acetate-Induced	Shen et al. (2024)	Lead Acetate Model SD Rats	EUE (100 mg/kg, 60 days)	↑ BMD, OPG/RANKL; protected against bone loss
Retinoic Acid-Induced	Xiong and Zhao (2020b), Feng, 2009; Zhao and Chen (2019)	Retinoic Acid-Induced Rats	Salt-processed Eucommia (1.5–6 g/kg, 4 w)	↑ BMD; modulated bone growth factors (BMP-2, VEGF) and cytokines (TNF-α, IL-6)

Abbreviations: ↑, increased or upregulated; ↓, decreased or downregulated; ALP, alkaline phosphatase; AR, androgen receptor; BMP2, bone morphogenetic protein 2; BMD, bone mineral density; BMC, bone mineral content; BMSCs, bone marrow mesenchymal stem cells; BV/TV, bone volume/tissue volume; Conn.D, connectivity density; DOP, diabetic osteoporosis; E2, oestradiol; EUE, *eucommia ulmoides* extract; FABP, fatty acid-binding protein; GH, growth hormone; GIOP, glucocorticoid-induced osteoporosis; IGF-1, insulin-like growth factor 1; IL-6, interleukin 6; OCN, osteocalcin; OPG, osteoprotegerin; OPN, osteopontin; OVX, ovariectomised; PINP, procollagen type I N-terminal propeptide; RANKL, receptor activator of nuclear factor κB ligand; Runx2, runt-related transcription factor 2; SD, sprague dawley; Tb.Th, trabecular thickness; Tb.N, trabecular number; Tb.Sp, trabecular separation; TGF-β1, transforming growth factor beta 1; TRACP, tartrate-resistant acid phosphatase.

### 3.4 The effects of *Eucommia ulmoides* leaves on osteoporosis

The osteoprotective potential of *Eucommia ulmoides* leaves (EUL) was supported by extensive *in vitro* and *in vivo* evidence. Early work showed that methanolic and acetone extracts stimulated collagen synthesis in a low-protein rat model, an effect attributed to the iridoid glycosides geniposidic acid and aucubin, thereby providing a biochemical basis for their traditional bone-strengthening properties (Li et al., 1998). In osteoblast-like MG-63 cells, isolated EUL compounds increased proliferation and ALP activity, with two constituents exhibiting the strongest osteogenic effects (Rao, 2004). EUL extract further promoted osteogenic commitment and suppressed adipogenesis in goat BMSCs, indicating regulation of lineage differentiation (Chen and Luo, 2009).

Active Component I isolated from EUL enhanced osteoblast differentiation and OPG secretion in a dose-dependent manner, while also stimulating renal OPG production. Co-culture assays demonstrated amplified osteogenic responses, suggesting multitarget regulation of bone metabolism through both skeletal and renal pathways (Cai, 2008). Additional studies reported that aqueous EUL extract increased osteoblast proliferation, differentiation, and mineralisation while reducing apoptosis, partly via activation of Wnt/β-catenin signalling. Extracts prepared through combined ethanol–water extraction and enzymatic hydrolysis also demonstrated notable osteogenic enhancement and synergised with Caltrate (Guan et al., 2021; Ma et al., 2024).

*In vivo*, EUL consistently mitigated oestrogen-deficiency bone loss. Total leaf extract improved bone metabolism and markedly increased femoral and tibial BMD in OVX rats (Rao, 2014). Ethanol extracts likewise increased bone density and serum oestradiol in diabetic and OVX models, suggesting an oestrogen-like effect (Zhang, 2003). Long-term EUL administration improved BMD, trabecular structure, and biochemical markers of bone turnover, with effects comparable to those of oestrogen treatment (Dai, 2012). Further studies showed that ethanol extracts reduced inflammatory cytokines, enhanced oestradiol secretion, and improved bone metabolic indices, confirming broad protective activity against post-menopausal osteoporosis (Liu, 2018; Du et al., 2023; Bai, 2008; Hu and Wang, 2002).

Increasing attention has been given to total flavonoids from EUL (TFEL). TFEL improved peak bone mass in young rats and strengthened trabecular structure, while reducing TRACP-5b. Network pharmacology implicated calcium-signalling, VEGF, IL-17, and NF-κB pathways in these effects (Zhang Y. et al., 2024). TFEL also improved bone microarchitecture and modulated gut microbiota in OVX rats, indicating both skeletal and systemic benefits (Yin et al., 2025). Water-extracted EUL enhanced BMD, bone strength, and reduced body weight in OVX rats, suggesting potential utility for managing both osteoporosis and menopause-associated metabolic changes (Zhang et al., 2012).

The gut–bone axis has emerged as a complementary therapeutic target. Aqueous EUL extract increased gut microbial diversity, elevated short-chain fatty acids, and improved BMD in ageing mice, demonstrating dual regulation of gut health and bone metabolism (Zhang Y. W. et al., 2024; Zhao X. et al., 2020).

The collective findings support a multifaceted osteoprotective profile for EUL. The key outcomes are summarised in Table 3.

**TABLE 3:** Experimental evidence for osteoprotective effects of Eucommia ulmoides leaf extracts.

Category	References	Model	Treatment (Dosage/Duration)	Major findings
*In Vitro* Studies				
Osteoblast and BMSC Models	Chen and Luo (2009)	MG-63 osteoblast-like cells	ELE (Compounds I–IV; 10^−4^–10^–8^ g/L; 4 days)	Compounds II and III potently ↑ cell proliferation and ALP activity
​	Cai (2008)	Goat BMSCs	ELE (10^−4^–10^–6^ g mL^-1^; 2 w)	↑ ALP activity and mineralised nodules; ↓ adipogenic differentiation
​	Ma et al. (2024)	MC3T3-E1 osteoblasts	ELE (0.05–0.35 mg/mL; 24 h)	↑ pre-osteoblast proliferation and mineralisation (↑ Col5a2, Runx2, Sox4, Bmp-4)
​	Ma et al. (2024)	Neonatal rat osteoblasts	ELE (10^–8^ mg/mL; 3 days)	Promoted osteoblast differentiation (↑ ALP, collagen I, BMP-2, Runx-2)
Multi-Target Mechanisms	Guan et al. (2021)	Neonatal rat osteoblasts; Kidney cells	Active Component I (10^−4^–10^–6^ g/mL; 96 h)	↑ ALP; ↑ OPG (osteoblasts and kidney); dual regulation of bone metabolism
*In Vivo* Studies				
OVX Model: Bone Density and Strength	Liu (2018)	OVX SD rats	Leaf ethanol extract (10 g/kg/d; 16 w)	↑ BMD, trabecular number; maintained bone metabolic balance
​	Zhang et al. (2024b)	OVX SD rats	ELE (1.25%–5% diet; 12 w)	↑ BMD, bone strength (Max-load, -stress, -strain); ↓ bone resorption markers
​	Zhang (2003)	OVX SD rats	ELE (1.5 g/kg/d; 90 days)	Improved bone metabolism; ↑ BMD (femur and tibia)
​	Zhang et al. (2014)	OVX SD rats	Leaf ethanol extract (6 g/kg; 90 days)	↑ femur weight, tibial bending strength, serum ALP.
OVX Model: Hormonal and Anti-inflammatory	Dai (2012)	Diabetic-OVX Wistar rats	ELE (6 g/kg/d; 8 w)	↑ femoral bone density and serum oestradiol; exerted oestrogen-like effects
​	Du et al. (2023)	OVX SD rats	Ethanol extract (6 g/kg/d; 8 w)	↑ BMD, E2; ↓ bone turnover markers, IL-6, TNF-α
​	Hu and Wang (2002), Zhang et al. (2024a)	OVX Wistar rats	Ethanol extract/Fractions (6 g/kg/d; 12 w)	↑ BMD, E2; ↓ IL-6; low-polarity fractions enhanced osteogenesis
OVX Model: Signalling Pathways	Bai (2008)	OVX SD rats	Eucommiae Folium granules (1.30–5.20 g/kg/d; 12 w)	↑ BMD; activated Wnt/β-catenin signalling (↑ Wnt3a, β-catenin; ↓ DKK1)
Other *In Vivo* Models				
Peak Bone Mass and Microarchitecture	Yin et al. (2025)	Normal young SD rats	TFEL (50–200 mg/kg; 13 w)	↑ peak bone mass, BMD, BV/TV, Tb.Th, Tb.N; improved trabecular structure
Bone and Gut Microbiota	Zhang et al. (2012)	OVX SD rats	TFEL (50–200 mg/kg; 13 w)	↑ BMD, BV/TV, Tb.Th, Tb.N; ↓ bone turnover; modulated gut microbiota
​	Li et al. (2011)	Senescent SAMP6 mice	ELE (1.5–3.0 g/kg; 12 w)	↑ BMD; improved gut microbiota (↑ L. bulgaricus, SCFAs)
Bone Matrix Formation	Rao (2004)	Low-protein diet rats	Geniposidic acid and Aucubin (25–100 mg/kg; 4 w)	Enhanced bone health via ↑ collagen synthesis

Abbreviations: ↑, increased or upregulated; ↓, decreased or downregulated; ALP, alkaline phosphatase; BMD, bone mineral density; BMP-2/4, bone morphogenetic protein 2/4; BMSC, bone marrow mesenchymal stem cell; BV/TV, bone volume/tissue volume; ELE, eucommia ulmoides leaf extract; OPG, osteoprotegerin; OVX, ovariectomised; RANKL, receptor activator of nuclear factor-κB ligand; Runx2, runt-related transcription factor 2; SCFAs, short-chain fatty acids; SD, Sprague-Dawley; Tb.Th, trabecular thickness; Tb.N, trabecular number; TFEL, total flavonoids from Eucommia ulmoides leaves.

### 3.5 Bioactive compounds from EU and their role in osteoporosis prevention and treatment

Multiple compounds derived from EU were investigated for their potential in osteoporosis prevention and treatment. These included total glycoside and lignans extracts, along with specific bioactive constituents such as aucubin, geniposide (GEN), 5-(hydroxymethyl)-2-furaldehyde (5-HMF), pinoresinol diglucoside (PDG), and rutin. Several derivatives, including Ser-Asp-Ser-Ser-Asp peptide-geniposidic acid conjugate (SGPA), were also examined.

Total glycosides from EU seed (TGEUS) were shown to enhance BMD, trabecular bone microstructure, and bone strength in growing female rats. These effects were attributed to aucubin, which stimulated osteoblast proliferation and suppressed osteoclast activity (Li et al., 2011). In OVX rats, TGEUS preserved trabecular bone by inhibiting the Notch signalling pathway and elevating osteogenic markers such as osterix, OCN, and RUNX2 (Zhou et al., 2021).

Total flavonoids from EU bark significantly promoted osteoblast proliferation *in vitro* (Xiao, 2008). In glucocorticoid-induced osteoporotic mice, EU seed glycosides were found to mitigate weight loss and improve femoral biomechanical properties (Li et al., 2010). Aucubin counteracted smoking-induced bone loss in rats by normalising bone turnover markers and improving trabecular parameters (Liu, 2012). Similarly, total flavonoids from EU cortex (TFE) reduced bone resorption and enhanced bone formation in OVX rats, demonstrating dose-dependent efficacy (Li S. H. et al., 2018).

In MC3T3-E1 osteoblastic cells, quercetin, geniposide, and aucubin were shown to enhance both proliferation and differentiation in a time- and concentration-dependent manner (Mou, 2015). An ethanol extract of EU leaves increased bone strength and serum ALP activity in OVX rats, comparable to alfacalcidol (Chen, 2015). Total lignans from EU bark were reported to prevent bone loss in OVX rats and exhibited dual activity by promoting osteoblast differentiation and inhibiting osteoclast formation *in vitro* (Zhang et al., 2014).

Aucubin, the most studied iridoid glycoside from EU, enhanced osteoblast differentiation through upregulation of ALP, collagen I, OCN, OPN, and BMP-2, mediated by Smad, MAPK, and Akt/mTOR pathways (Li Y. et al., 2018). In OVX mice, it suppressed osteoclastogenesis via MAPK and NF-κB signalling and promoted type H vessel formation (Li et al., 2022). Aucubin also facilitated angiogenesis via VEGF and angiopoietin-Tie pathways while inhibiting osteoclast activity (He et al., 2023b). Moreover, it was shown to alleviate GIOP by upregulating osteogenic markers and modulating arachidonic acid metabolism (Huang et al., 2024; Wang H. et al., 2024). In a recent study, aucubin was found to inhibit ferroptosis and promote osteogenesis in BMSCs from postmenopausal osteoporosis patients via the BMP-2/SMAD pathway (Zheng Y., 2025).

GEN was reported to activate autophagy via the GLP-1R/PI3K/AKT/mTOR pathway, protecting against GIOP (Huang et al., 2022). It also attenuated oxidised LDL-induced osteoblast apoptosis through NRF2/NF-κB signalling (Xiao et al., 2022; Xiao Y. et al., 2023). Quercetin promoted osteogenesis and reduced oxidative stress and apoptosis via the Nrf2/HO-1 pathway (Xiao J. et al., 2023). Rutin enhanced BMD and trabecular structure in OVX rats and promoted BMSC mineralisation through autophagy and Akt/mTOR signalling (Xiao et al., 2019).

5-HMF stimulated osteogenic differentiation and suppressed adipogenesis in BMSCs (Tan et al., 2014). PDG was shown to improve BMD and calcium levels while reducing inflammation and RANKL/OPG ratio in dexamethasone-induced osteoporosis (Zuo et al., 2024; Zhang et al., 2016). The polysaccharide EuOCP3 improved bone microstructure and reduced oxidative stress via ERK/JNK/Nrf2 pathways (Song et al., 2023), and also promoted osteoblast differentiation via ERK/BMP-2/SMAD signalling (Song et al., 2024).

Chlorogenic acid enhanced osteoblast activity, inhibited osteoclast formation, and reversed bone loss in OVX rats (Ho et al., 2024). Kaempferol promoted osteogenic differentiation of BMSCs at low concentrations, though higher levels impaired proliferation (Li et al., 2024b). A targeted conjugate, SGPA, enhanced osteoblast activity and bone formation in OVX mice via the farnesoid X receptor–RUNX2 pathway (Liu et al., 2023).

In a comparative study, kaempferol exhibited the strongest anti-osteoporotic effect among EU flavonoids, followed by rutin and quercetin (Yuan, 2018). Total flavonoids from EU leaves also modulated gut microbiota and improved bone density in OVX rats (Zhang, 2022). Pinoresinol diglucoside and pinoresinol stimulated osteoblast proliferation and differentiation, with the latter also modulating the OPG/RANKL ratio (Hu, 2018). In perimenopausal rats, total flavonoids restored hormonal balance and upregulated estrogen and androgen receptors (Tian et al., 2018). β-Sitosterol was found to enhance bone formation via β-catenin signalling in OVX mice (Jiang, 2024). 5-HMF improved bone density and inflammation markers, partially by restoring gut microbiota composition (Liu, 2024). EU flavone ameliorated bone loss in OVX rats via the AMPK/PAK2 pathway and reduced oxidative stress (Lang et al., 2025).

The key outcomes of the studies presented in this section are summarised in Table 4, 5.

**TABLE 4:** *In vitro* studies of bioactive compounds from *Eucommia ulmoides* on osteoporosis.

Compound	References	Cellular model	Dosage/Duration	Major findings and Proposed mechanism
Aucubin (AU)	Li et al. (2022)	MG63 osteoblasts	1–5 μM; 24–48 h	Enhanced osteoblast differentiation via BMP2/MAPK/Akt pathways (↑ Smad1/5/8, JNK, p38, Akt/mTOR)
​	Wang et al. (2024b)	MC3T3-E1 osteoblasts	1–5 μM; 24 h	Upregulated osteogenic markers (↑ BMP2, OPN, RUNX2, COL-1)
​	Huang et al. (2022)	hBMSCs (from OP patients)	5–20 μM; 24–72 h	Protected against oxidative stress and promoted osteogenesis (↓ ROS, MDA, Fe2+; ↑ SOD, ALP, BGLAP)
​	Huang et al. (2024)	HUVECs	3.13–50 μM; 48 h	Promoted angiogenesis (↑ VEGFR2, MEK, ERK, Bcl2/Bax ratio)
​	He et al. (2023b)	RAW264.7 cells	1–5 μM; 1–6 days	Inhibited osteoclastogenesis via MAPK/NF-κB pathway (↓ NFATc1, CTSK, DC-STAMP, c-Fos)
5-HMF	Zhang et al. (2016)	BMSCs (SD rats)	0.05–0.20 μg/mL; 7–21 days	Promoted osteoblastogenesis and inhibited adipogenesis (↑ ALP, COL1α1, OCN, OPN; ↓ PPARγ, FABP4, C/EBPα)
Rutin	Tan et al. (2014)	BMSCs (SD rats)	Unspecified; 3 weeks	Enhanced BMSC mineralisation via autophagy (↓ FNDC1, p62; ↑ LC3-II/LC3-I ratio)
TGEUS	Xiao (2008)	ADSCs (human)	1–10 μM; 48 h	Stimulated osteogenic differentiation (↑ OSX, OCN, RUNX2)
Geniposide (GEN)	Xiao et al. (2023a)	MC3T3-E1 cells	25 μM; 48 h	Reduced oxidative damage in osteoblasts (↓ ER stress)
EuOCP3	Ho et al. (2024)	MC3T3-E1 cells	10–20 μg/mL; 21 days	Promoted osteoblast differentiation via ERK/BMP-2/SMAD pathway (↑ RUNX2, OCN)
Kaempferol	Liu et al. (2023)	BMSCs (human)	0.1–50 μM; 24 h	Enhanced osteogenic differentiation (↑ ALP, Collagen I, RUNX2, OPN; ↓ CAV-1)
Pinoresinol and Derivatives	Tian et al. (2018)	MC3T3-E1 cells	10^−7^–10^3^ μg/L; 48–72 h	Promoted osteogenesis; Pinoresinol showed stronger anti-resorptive potential by ↑ OPG and ↓ RANKL
Multi-Compound Study	Chen (2015)	MC3T3-E1 cells	10^−2^–10^–6^ mmol/L; 5 days	Quercetin, geniposide, and aucubin enhanced osteoblast proliferation and ALP activity in a dose- and time-dependent manner

Abbreviations: ↑, increased or upregulated; ↓, decreased or downregulated; ADSCs, Adipose-derived stem cells; ALP, alkaline phosphatase; BMSC, bone marrow mesenchymal stem cell; BMP-2, Bone morphogenetic protein 2; COL-I, Collagen type I; ERK, Extracellular signal-regulated kinase; HUVECs, Human umbilical vein endothelial cells; OCN, osteocalcin; OPN, osteopontin; OPG, osteoprotegerin; PPARγ, Peroxisome proliferator-activated receptor gamma; RANKL, Receptor activator of nuclear factor-κB ligand; ROS, reactive oxygen species; RUNX2, Runt-related transcription factor 2; TGEUS, total glycosides from *eucommia ulmoides* seed.

**TABLE 5:** *In vivo* studies of bioactive compounds from *Eucommia ulmoides* on osteoporosis.

Compound/Extract	References	Animal model	Dosage/Duration	Major findings
Aucubin (AU)	He et al. (2023b)	OVX BALB/c mice	5 mg/kg; 1 month	Inhibited osteoclast maturation (↓ MAPK/NF-κB), promoted type H vessels, improved microarchitecture
​	Wang et al. (2024b)	DEX-induced OP SD rats	15 mg/kg; 6 weeks	↑ Osteogenic markers (BMP2, RUNX2), improved bone remodelling and microarchitecture
​	Zheng (2025b)	DEX-induced OP Balb/c rats	5–20 mg/kg; 4 weeks	Modulated arachidonic acid metabolism, improved BMD and trabecular structure
​	Huang et al. (2022)	OVX SD rats	30 mg/kg; 2 months	Promoted osteogenesis, inhibited ferroptosis, and reduced inflammation
​	Li et al. (2018a)	Smoking-induced OP SD rats	300 mg/kg/day; during exposure	Protected against bone loss, improved BMD and trabecular parameters
5-HMF	Lang et al. (2025)	OVX SD mice	100 mg/kg/day; 12 weeks	Improved BMD, reduced inflammation, and modulated gut microbiota
β-Sitosterol	Liu (2024)	OVX C57BL/6J mice	5 mg/kg/day; 8 weeks	Enhanced osteogenesis via β-catenin, preserved trabecular structure
Flavonoids (Mixed/Comparative)	Zhang (2022)	OVX SD rats	Quercetin, Kaempferol, Rutin (50 mg/kg/d; 12 w)	Kaempferol showed strongest bone-protective effect, all improved BMD and microarchitecture
​	Jiang (2024)	Perimenopausal Wistar rats	Total leaf flavonoids (50–200 mg/kg; 30 days)	Corrected sex hormone imbalance and improved bone metabolic markers
​	Yi (2024)	OVX SD rats	Eucommia flavone (EF) (40–160 mg/kg/d; 2 w)	Activated AMPK/PAK2 signalling, improved bone metabolism and microstructure
Total Glycosides/Flavonoids	Zhou et al. (2021)	Growing SD rats	TGEUS (400 mg/kg; 12 w)	Enhanced bone density and microarchitecture via anabolic/anti-resorptive effects
​	Mou (2015)	OVX SD rats	TFE (50–200 mg/kg/d; 12 w)	Promoted bone formation, reduced resorption, and protected bone tissue
​	Liu (2012)	GIOP ICR mice	Eucommia seed glycosides (55–220 mg/kg/d; 8 w)	Improved femoral biomechanical strength and alleviated osteoporosis
​	Hu (2018)	OVX SD rats	Total leaf flavonoids (200 mg/kg/d; 13 w)	Improved BMD, microarchitecture, and modulated gut microbiota
Total Lignans (TL)	Li et al. (2018b)	OVX SD rats	TL (20–80 mg/kg; 16 w)	Exerted dual regulatory effect on bone remodelling, prevented bone loss
Pinoresinol Diglucoside (PDG)	Song et al. (2023)	DEX-induced OP Wistar rats	PDG (10–40 mg/kg; 8 w)	Attenuated GIOP by promoting bone formation and inhibiting resorption
​	Arksey and O'Malley (2005)	Zebrafish larvae	PDG (0.75–3 μM; 7–9 days)	Mitigated glucocorticoid-induced skeletal defects via Wnt activation
Rutin	Tan et al. (2014)	OVX SD rats	Rutin (10 mg/kg; 10 w)	Improved trabecular structure via autophagy
Geniposide (GEN)	Xiao et al. (2022)	DEX-induced OP SD rats	GEN (50–100 mg/kg; 4 m)	Prevented bone loss via GLP-1R/PI3K/AKT/mTOR-mediated autophagy
​	Xiao et al. (2023a), Xiao et al. (2023b)	DEX/HFD-induced OP rats	GEN (50–100 mg/kg; 4–6 m)	Protected against bone loss via NRF2-mediated cytoprotection and NF-κB suppression
Targeted Conjugate	Yuan (2018)	OVX C57BL/6J mice	SGPA (20 mg/kg; 4 w)	Enhanced bone formation via FXR-RUNX2 pathway activation
Polysaccharide	Song et al. (2024)	DEX-induced OP C57BL/6 mice	EuOCP3 (100–300 mg/kg; 49 days)	Enhanced bone formation via ERK/BMP-2/SMAD and gut-bone axis modulation
Chlorogenic Acid	Li et al. (2024b)	OVX SD rats	Chlorogenic acid (25–100 mg/d; 8 w)	Promoted osteoblast proliferation, enhanced mineralisation, reduced bone loss

Abbreviations: ↑, increased or upregulated; ↓, decreased or downregulated; BMD, bone mineral density; BV/TV, Bone volume/tissue volume; DEX, dexamethasone; GIOP, Glucocorticoid-induced osteoporosis; HFD, High-fat diet; OVX, ovariectomised; Tb.N, trabecular number; Tb.Th, Trabecular thickness; TFE, total flavonoids from eucommia cortex; TGEUS, total glycosides from *eucommia ulmoides* seed.

### 3.6 Clinical studies on *Eucommia ulmoides* in osteoporosis management

Clinical investigations examining EU-based formulations have reported encouraging outcomes in patients with osteoporosis. A serum metabolomics study found that Quanduzhong capsules reversed alterations in amino acid metabolism associated with osteoporosis, identifying potential biomarkers such as kynurenine and arginine (Yi, 2024). In a comparative clinical trial, combining Quanduzhong capsules with alendronate produced a higher overall response rate, faster symptom relief, and greater improvements in BMD and bone resorption markers compared with alendronate alone (Li J. et al., 2024).

EU granules have also been shown to reduce low back pain, suppress bone resorption markers, and improve BMD in patients with primary osteoporosis, with effects comparable to alfacalcidol (Zhang, 2009b). Similarly, the addition of Quanduzhong capsules to calcium supplementation significantly enhanced BMD and overall clinical efficacy compared with calcium alone (Tang, 2012).

In patients undergoing percutaneous vertebroplasty for osteoporotic vertebral compression fractures, Quanduzhong capsules improved bone metabolism, reduced pain, and decreased the incidence of bone cement leakage (Sun and Wu, 2022). Comparable benefits were observed with EU capsules combined with calcium, which enhanced BMD and reduced bone resorption markers post-surgery (Wu et al., 2024). Quanduzhong capsules also improved bone density, immune markers, and bone metabolism in patients after lower limb fracture surgery (Wang and Sun, 2022). Furthermore, adjunct use of Quanduzhong capsules following vertebroplasty was associated with a lower risk of refracture and improved spinal alignment (Cai, 2021).

A combination of EU granules and salmon calcitonin improved BMD and Wnt pathway-related markers in primary osteoporosis patients (Wang, 2018). Similarly, a herbal regimen containing Quanduzhong capsules and Honghua Xiaoyao tablets significantly increased BMD and oestradiol levels in postmenopausal women with osteoporosis (Wang and Chu, 2014).

Collectively, the clinical findings reported in the identified literature suggest that EU-based formulations may enhance BMD, alleviate pain, and improve bone metabolic indicators across various osteoporosis-related conditions. Table 6 presents the key characteristics of the clinical studies on EU formulations included in this review.

**TABLE 6:** Clinical studies on *Eucommia ulmoides* formulations in osteoporosis management.

References	Patient population	Intervention and dosage	Duration	Major outcomes
Li et al. (2024c)	30 OP patients vs. 30 controls	QDZ (3 g t.i.d.)	16 weeks	Reversed amino acid metabolic changes associated with OP.
Zhang (2009b)	98 elderly OP patients	Alendronate (10 mg/d) + QDZ (2 caps b.i.d.)	3 months	↑ BMD, ↓ bone resorption markers, superior to alendronate alone
Tang (2012)	68 primary OP patients with LBP	Eucommia granules (1 g t.i.d.) vs. alfacalcidol	6 months	↓ Pain, ↑ bone formation markers, ↓ resorption markers, ↑ BMD.
Sun and Wu (2022)	80 primary OP patients	QDZ (4–6 caps b.i.d.) + Calcium	3 months	↑ Lumbar and femoral neck BMD; total effective rate 92.5%
Wu et al. (2024)	80 OVCF patients post-PVP	QDZ (1.2 g/d) + Calcium D	3 months	↑ BMD, ↓ pain, ↓ bone turnover markers, ↓ bone cement leakage
Wang and Sun (2022)	60 OVCF patients post-PVP	Eucommia capsules (2.88 g/d) + Calcium D3	3 months	Improved pain, BMD, bone metabolism, and reduced cement leakage
Cai (2021)	60 post-op OP fracture patients	QDZ (6 caps b.i.d.) + Vit D–Ca	3 months	↑ BMD, improved bone turnover and immune function
Wang (2018)	OP vertebral fracture patients	PVP + QDZ (2–3 caps b.i.d.)	6 months	↑ BMD, improved spinal alignment, relieved pain, reduced refracture risk
Wang and Chu (2014)	116 primary OP patients	Std therapy + Eucommia granules + Calcitonin	6 months	↑ BMD, ↓ SOST/DKK-1, improved symptoms and Wnt pathway markers
Liang et al. (2017)	75 postmenopausal OP women	QDZ + Honghua Xiaoyao tablets	6 months	↑ BMD and E2, ↓ bone resorption markers, superior efficacy

Abbreviations: ↑, increased; ↓, decreased; b.i.d., twice daily; BMD, bone mineral density; LBP, low back pain; OP, osteoporosis; OVCF, osteoporotic vertebral compression fracture; PVP, percutaneous vertebroplasty; QDZ, Quanduzhong capsule/capsules; t.i.d., three times daily.

In terms of quality, the clinical trials included have generally low-to-moderate methodological quality and a high risk of bias. For randomisation, most studies cite the use of “random number tables” for sequence generation (Li J. et al., 2024; Sun and Wu, 2022; Wu et al., 2024; Wang, 2018), indicating true randomisation. However, a significant number merely state “randomised” without specifying the method. Importantly, none of the trials describe allocation concealment mechanisms, leaving them susceptible to selection bias.

For performance and detection bias, none of the trials report the use of placebos or blinding of participants, personnel, or outcome assessors. This is particularly problematic for subjective outcomes, like pain relief (VAS scores), which are frequently used across these trials. The lack of blinding inflates the risk of placebo effects and observer bias. For attrition and reporting Bias, Intention-to-Treat (ITT) analysis is largely absent. Most studies analyse only those who completed the treatment, potentially overestimating efficacy.

In conclusion, while these articles provide consistent positive evidence for the efficacy of EU preparations, significant methodological limitations exist. The evidence should be interpreted with caution, and higher-quality, double-blind RCTs are necessary to confirm these findings.

## 4 Discussion

The comprehensive analysis presented in this review substantiates the therapeutic potential of EU in osteoporosis management, reinforcing its historical application in TCM for enhancing bone health (Pan et al., 2014; Zhang et al., 2012). Osteoporosis, a prevalent skeletal disorder characterised by diminished BMD and an elevated fracture risk, poses a significant public health challenge, particularly among ageing populations. EU exhibits a multifaceted pharmacological profile that effectively addresses these pathological manifestations by modulating bone remodelling, enhancing osteoblastic bone formation while simultaneously attenuating osteoclastic resorption (Zhang et al., 2009; Zhang et al., 2012; Li et al., 2011; Huang et al., 2024).

### 4.1 Bone protective mechanism of EU

**FIGURE 2:**
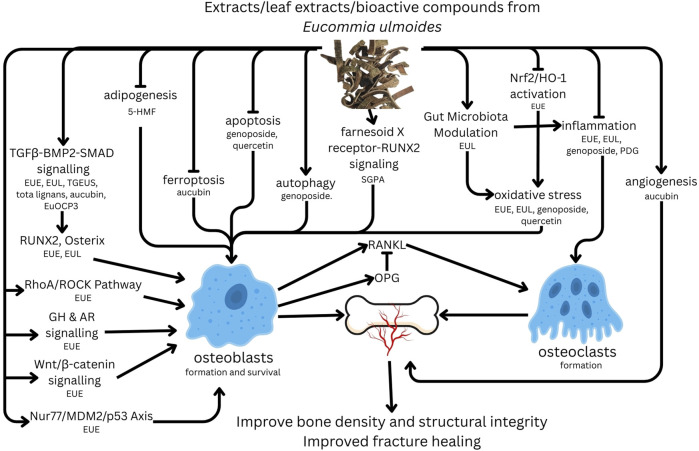
Mechanism of action of *Eucommia ulmoides* extracts (EUE), leaf extract (EUL) and bioactives on bone. Notes: pointy arrow, stimulation; flat arrow, inhibition. Abbreviations: 5-HMF, 5-hydroxymethylfurfural; BMP2, bone morphogenetic protein 2; EuoCP3, *Eucommia ulmoides* polysaccharide 3; GH, growth hormone; HO-1, haem oxygenase-1; Nrf2, nuclear factor erythroid 2–related factor 2; OPG, osteoprotegerin; RANKL, receptor activator of nuclear factor κB ligand; Runx2, runt-related transcription factor 2; PDG, pinoresinol diglucoside; TGEUS, total glycosides from *Eucommia ulmoides* seed; TGF-β1, transforming growth factor beta 1; SGPA, SDSSD (an osteoblast-targeting peptide)-geniposidic acid conjugate.

EU and its bioactive constituents exert potent osteogenic effects through multiple molecular pathways. The extracts have been shown to upregulate key osteogenic transcription factors, including RUNX2 and Osterix, which are essential for the differentiation of mesenchymal stem cells into osteoblasts (Lee et al., 2016; Guan et al., 2021; Zhou et al., 2021; Liu et al., 2023). Activation of the BMP/SMAD and Wnt/β-catenin signalling pathways further reinforces osteoblast commitment and extracellular matrix mineralisation (Mai et al., 2024). Notably, EU-derived compounds, such as aucubin and geniposide, stimulate collagen type I synthesis and ALP activity, both of which are critical for bone matrix maturation (Li Y. et al., 2018; Huang et al., 2024; Liang et al., 2017). Additionally, the PI3K/Akt/mTOR pathway is modulated by geniposide, promoting osteoblast survival and differentiation (Huang et al., 2022). These mechanisms have been consistently demonstrated in various osteoporosis models, including PMOP and GIOP (Zhou, 2016; Zhang et al., 2009). The key signalling pathways modulated by EU extracts and EU’s bioactive compounds are summarised in Figure 2.

In parallel, EU extracts effectively inhibit excessive bone resorption by targeting osteoclast differentiation and activity. A crucial mechanism involves the modulation of the RANKL/OPG axis, wherein EU components downregulate RANKL expression while upregulating OPG, thereby fostering an anti-osteoclastic microenvironment (Lee et al., 2016; Qi et al., 2019; Yin et al., 2025). At the molecular level, aucubin and flavonoids inhibit NF-κB and MAPK signalling, both critical for osteoclastogenesis (Li et al., 2022). Furthermore, EU downregulates osteoclast-specific markers such as TRACP5b and cathepsin K, while attenuating pro-inflammatory cytokines (e.g., IL-6, TNF-α) that promote bone resorption (Yang et al., 2022; Zhang Y. et al., 2024). The antioxidant properties of EU, mediated via the Nrf2/HO-1 pathway, further contribute to its anti-resorptive effects by reducing oxidative stress-induced bone loss (Shen et al., 2024; Xiao J. et al., 2023).

Beyond the direct effects on bone cells, the EU modulates systemic factors that influence bone health. For instance, EU leaf extracts regulate the gut–bone axis by promoting beneficial microbiota (e.g., *Lactobacillus sp*) and increasing short-chain fatty acid production, thereby enhancing calcium absorption and bone density (Zhao X. et al., 2020). Additionally, the EU plays a role in mineral homeostasis by maintaining a balance in serum calcium and phosphorus levels (Qi et al., 2019; Yang et al., 2022). In GIOP, EU counteracts bone suppression by activating BMP-2 and androgen receptor signalling (Zhou, 2016).

These pleiotropic actions highlight EU’s potential as a therapy for diverse osteoporosis aetiologies, modulating both osteogenic activities of osteoblasts and antiresorptive activities of osteoclasts. In contrast with standard anti-osteoporosis pharmacotherapies, which act distinctly on either the bone formation or resorption process, the dual actions of EU suggest a more balanced, modulatory effect. Based on the preclinical evidence summarised in Tables 2–5, the evidence for its anabolic effects appears slightly more robust and consistently reported across various extracts and compounds (e.g., promoting Runx2/Osterix, activating Wnt/β-catenin and BMP/SMAD pathways). While it is clearly anti-resorptive (modulating RANKL/OPG, inhibiting NF-κB), this dual nature positions EU uniquely. For example, unlike pure anti-resorptive agents such as bisphosphonates, which can lead to over-suppression of bone turnover, EU offers a combined approach similar to newer anabolic/antiresorptive agents (like romosozumab), potentially leading to more favourable long-term bone quality outcomes by maintaining the coupling of bone formation and resorption.

### 4.2 Perspective on future research

Animal studies consistently demonstrate improvements in key bone health indicators, including increased bone volume fraction (BV/TV), trabecular number (Tb.N), and trabecular thickness (Tb.Th), alongside enhanced biomechanical properties (Zhang et al., 2009; Li et al., 2011; Xiao et al., 2019). However, the limited number of large-scale clinical studies underscores the need for rigorous human trials to validate these findings (Yi et al., 2024). Based on the potent effects demonstrated in preclinical models (Tables 4, 5), AU and GEN emerge as the most promising compounds for immediate translational study. Key remaining questions for these specific compounds include validating their bioavailability in human subjects and confirming their long-term safety profile. An ideal clinical trial design would focus on prevention in high-risk patient populations, such as postmenopausal women with osteopenia. Primary endpoints should include changes in P1NP and CTX-1 (bone turnover markers) over 6–12 months, followed by changes in BMD (spine/hip) over 12–24 months, with fracture risk as a long-term secondary endpoint. The comparator should be a calcium and/or vitamin D supplement.

The gap between preclinical and clinical evidence is a significant challenge for EU and traditional herbal medicines in general. Moving EU toward clinical use requires overcoming significant barriers, primarily concerning the standardisation of extracts. The efficacy of EU extracts can vary widely based on the specific plant part used (bark vs. leaf), geographical origin, and the extraction methodology (aqueous vs. methanol). Establishing batch-to-batch consistency, as well as defining the optimal effective dose and compound fingerprint for a clinical product, are critical before clinical translation. Furthermore, designing placebo-controlled clinical trials for EU could be challenging, as EU extracts often have a distinct taste or smell, making blinding difficult. Future research must, therefore, prioritise the development of standardised, chemically characterised clinical-grade extracts before launching large-scale, high-quality human intervention studies.

In addition to these translational challenges, future investigations should also prioritise a more comprehensive understanding of the multi-target and multi-pathway pharmacology of EU. The diverse pharmacological mechanisms underlying the therapeutic effects of EU warrant continued investigation to elucidate its multi-target and multi-pathway interactions (Shao, 2022; Qin et al., 2024; Wang P. et al., 2023; Feng et al., 2022). Further research should focus on identifying the precise molecular pathways that regulate the intricate balance between osteoblast and osteoclast functions (Hansen et al., 2023; Xie et al., 2023; Wang F. et al., 2019). Importantly, pharmacokinetic and bioavailability studies of key bioactive compounds, particularly AU, GEN, and other lignans, are urgently needed, as their absorption, metabolism, tissue distribution, and clearance remain poorly characterised. Such studies will be essential for determining effective human dosages, optimising formulation strategies (e.g., nano-formulations, sustained-release preparations) and predicting potential herb–drug interactions. These factors significantly impact therapeutic efficacy (Yan et al., 2018; Huang Q. et al., 2021; Wang Z. et al., 2023).

From a safety and regulatory perspective, future evidence must extend beyond small cohorts. Exploring the safety profile and potential adverse effects of EU in larger and more diverse populations will be critical for its integration into clinical practice (Zhang Y. et al., 2024; Luo et al., 2020; Zeng et al., 2020). Regulatory considerations, including toxicological profiling, maximum permissible dose ranges, and compliance with pharmacopoeial standards, should be incorporated early in development to facilitate future approval as an evidence-based herbal intervention.

Finally, strengthening collaboration between traditional herbal medicine and modern pharmacological science will be essential. Such interdisciplinary efforts can accelerate the development of standardised extracts or novel formulations that maximise therapeutic benefits while ensuring safety, reproducibility and regulatory compliance (Huang L. et al., 2021; Chan et al., 2020).

### 4.3 Limitations of the current review

While this review provides a map of current evidence, several aspects merit consideration to inform future research directions. Variability in extraction techniques and dosage regimens across studies presents challenges for direct comparison and standardisation. Additionally, the preclinical data presented demonstrate significant heterogeneity in research protocols, including the use of different animal models (OVX, GIOP, senescence and high-fat diet), widely divergent dosages (e.g., 50–500 mg/kg for extracts), and varying treatment durations (from 6 weeks to 4 months). This lack of standardisation makes cross-study comparisons difficult and highlights the need for harmonised research protocols in the future. Although preclinical models offer valuable mechanistic insights, their translational relevance to human osteoporosis may be limited. Moreover, restricting the search to English-language publications and widely used international databases may have inadvertently excluded pertinent studies published in other languages or indexed in regional repositories. Formal statistical evaluation of publication bias and standardised risk-of-bias assessments, while beyond the present scope, could enhance methodological rigour in future reviews.

Notwithstanding these considerations, the present findings consolidate EU as a promising multi-target candidate in osteoporosis management. Future research should seek to integrate multilingual evidence sources, adopt established quality appraisal tools, and pursue well-designed clinical trials to support its safe and effective application in clinical practice.

## 5 Conclusion

EU demonstrates significant potential in osteoporosis management through its dual action of promoting osteoblast activity and inhibiting osteoclastogenesis. This effect is achieved via the upregulation of osteogenic markers such as Runx2, BMP-2, and collagen I, as well as modulation of the RANKL/OPG pathway. Extracts and bioactive constituents, including aucubin and geniposide, have been shown to improve bone mineral density and microarchitecture in various osteoporosis models, including PMOP, GIOP, and diabetic forms. These effects are mediated through the activation of signalling pathways such as BMP/SMAD, Wnt/β-catenin, and Nrf2/HO-1. Moreover, EU contributes to bone health by regulating oxidative stress and modulating the gut microbiota, highlighting its value as a multi-target therapeutic agent. Although current evidence is largely preclinical, the consistent osteoprotective outcomes underscore the need for further clinical studies to validate efficacy, safety, and optimal formulations. Overall, EU represents a promising natural candidate for the prevention and treatment of osteoporosis within an integrative medical framework.
